# Co-delivery of sorafenib and metformin from amphiphilic polypeptide-based micelles for colon cancer treatment

**DOI:** 10.3389/fmed.2022.1009496

**Published:** 2022-10-11

**Authors:** Xiaohui Zhang, Lanqing Cao, Guangmeng Xu, Hongyu He, Hongyu Zhao, Tongjun Liu

**Affiliations:** ^1^Department of General Surgery, The Second Hospital of Jilin University, Changchun, China; ^2^Department of Thyroid, Breast and Hernia Surgery, Affiliated Hospital of Inner Mongolia University for the Nationalities, Tongliao, China; ^3^Department of Pathology, The Second Hospital of Jilin University, Changchun, China; ^4^Operating Theater and Department of Anesthesiology, The Second Hospital of Jilin University, Changchun, China; ^5^Gastroenterology and Center of Digestive Endoscopy, The Second Hospital of Jilin University, Changchun, China

**Keywords:** colorectal cancer, drug delivery system, chemotherapy, micelles, tumor environment (TME)

## Abstract

Colorectal cancer (CRC) is a common clinical disease with a poor prognosis and a high recurrence rate. Chemotherapy is important to inhibit the post-surgical recurrence of CRC patients. But many limitations restrict the further application of chemotherapy. In this study, sorafenib (Sor) and metformin (Met) co-loaded poly(ethylene glycol)-block-poly(L-glutamic acid-*co*-L-phenylalanine) [mPEG-*b*-P(Glu-*co*-Phe)] micelles were developed. The characterizations, drug release, *in vivo* biodistribution, and pharmacokinetics of the micelles were analyzed. The treatment efficacy of the dual-drug loaded micelles was evaluated in a subcutaneous colon cancer mice model. Sor is a common molecular target agent that can inhibit the mitogen-activated protein kinase (MAPK) pathway to treat solid tumors. Met can also regulate the MAPK pathway and inhibit the expression of the phosphorylated extracellular signal-regulated kinase (p-ERK). Moreover, both Sor and Met play important roles in cell cycle arrest. The integration of these two drugs aims to achieve synergistic effects against colon cancer. The micelles can be targeted to cancer cells and possess longer blood circulation time. The two agents can be released rapidly in the tumor sites. The *in vivo* study showed that the micelles can prevent tumor progression by inhibiting the expressions of p-ERK and cyclin D1. This study indicated that the Sor/Met-loaded micelles are suitable for CRC treatment.

## Introduction

Colorectal cancer (CRC) threatens people's health seriously worldwide. Despite the advanced development in CRC diagnosis and surgical intervention, tumor recurrence tends to happen in lots of patients (1). Systematic chemotherapy is another method to extend the survival of CRC patients (2). However, the concentration of traditional chemotherapy agents within tumor sites is always not effective for tumor killing (3). In addition, patients with CRC are always intolerant of the side effects of systematic chemotherapy (4). As a result, achieving better therapeutic effects on CRC is important.

Nanotechnology, an emerging science, has promoted the development of pharmacy (5). The nanosized drug delivery systems can overcome the disadvantages of systematic chemotherapy (6). Nowadays, researchers are focusing on developing polymeric nanoparticles, such as vesicles (7, 8) and micelles (9, 10), for tumor therapy. The nanomaterials-based drug carriers not only protect the encapsulated agents during blood circulation but also increase the accumulation in the tumor site (11). Furthermore, co-drug-loaded nanoparticles to deliver combination therapy for CRC treatment have attracted more and more attention (12). Encapsulating chemotherapeutic agents with synergistic effects can increase the antitumor efficacy against CRC (13).

Sorafenib (Sor) can decrease the phosphorylated extracellular signal-regulated kinase (p-ERK) levels and block the mitogen-activated protein kinase (MAPK) pathway to inhibit tumor progression (14, 15). The MAPK pathway in tumor cell lines is associated with tumor development, including tumor growth, differentiation, and apoptosis (16, 17). ERK is a key component in the MAPK pathway, and tumor cell proliferation depends on p-ERK (14). In addition, Sor also exhibits anti-proliferative activity in tumors by inhibiting cyclin D1 expression (18). Sor is approved for the treatment of hepatoma clinically. Recent studies also showed that CRC patients may be benefited from Sor (19) and Sor could prevent the proliferation and metastasis of CRC cell lines (20). Metformin (Met), a safe hypoglycemic agent, has been proved of tumor inhibition effect (21), and can also inhibit the expression of p-ERK (22, 23) and cyclin D1 (24, 25). Therefore, we hypothesize that the integration of Sor and Met can increase the synergistic effects of CRC treatment. Delivering the two drugs while decreasing the side effects is crucial for tumor therapy.

**Figure 1:**
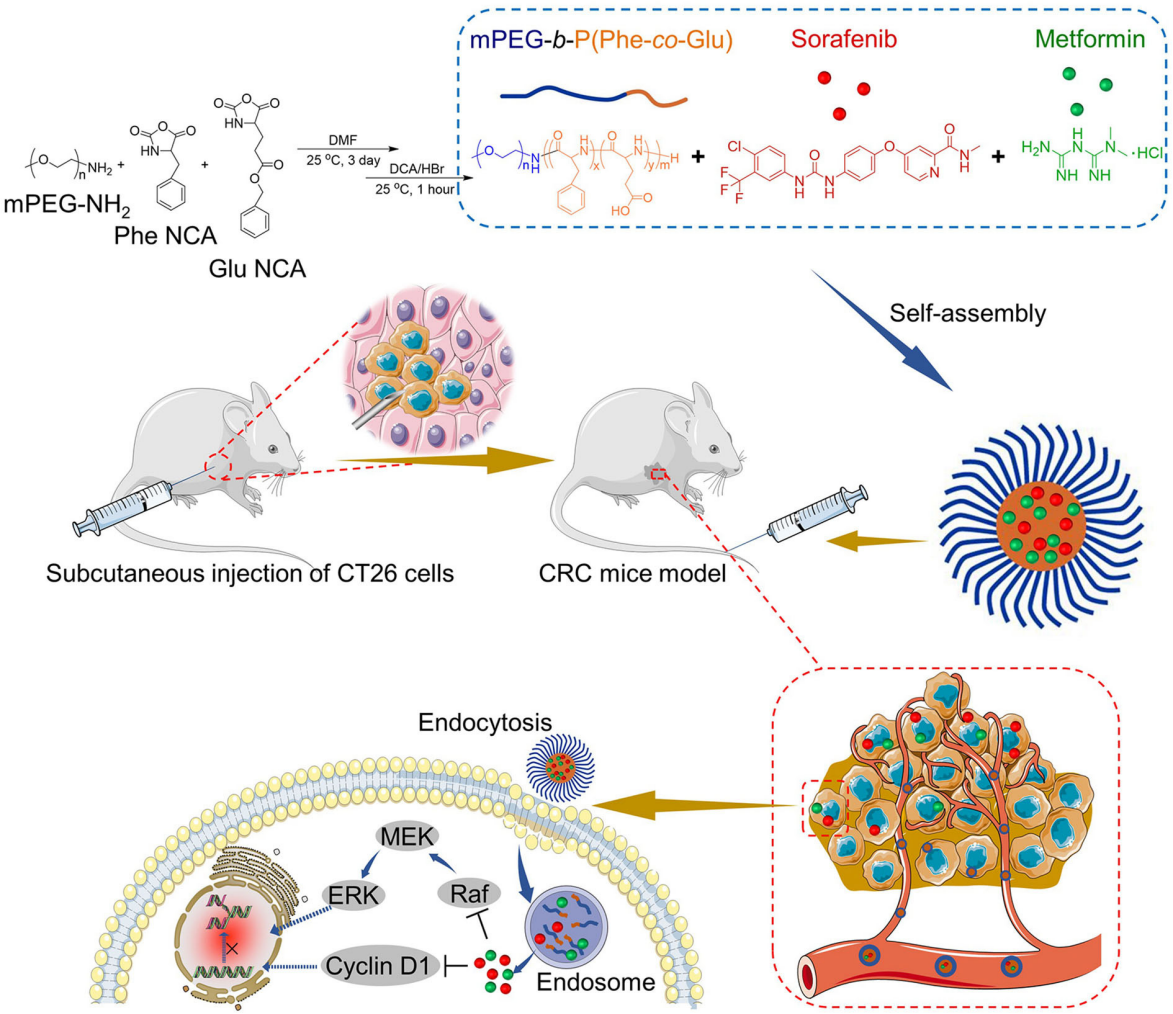
Synthesis of Met/Sor-loaded mPEG-*b*-P(Glu-*co*-Phe) micelles and their mechanisms of CRC prevention.

In this study, poly(ethylene glycol)-block-poly(L-glutamic acid-*co*-L-phenylalanine) [mPEG-*b*-P(Glu-*co*-Phe)] micelles were prepared, followed by the encapsulation of Sor and Met. Herein, the copolymers can be self-assembled, and different components of the copolymer possess different functions to deliver Sor and Met. The PEG shell mainly provides the protective effects for the loading agents. Glutamic acid units assist in electrostatic interaction between the glutamic acid carboxyl group and the Met amino group. Sor is hydrophobic and can be loaded into the nanocarrier by physical embedding. Phenylalanine units increase the hydrophobic/aromatic interaction within the inner core of micelles (6). The characteristics of the dual-drug-loaded micelles were analyzed *in vivo* and *in vitro*. A subcutaneous colon cancer mice model was applied to evaluate the treatment efficacy of the Sor and Met co-loaded micelles. Sor and Met were successfully delivered to the tumor sites. Sor and Met loaded mPEG-*b*-P(Glu-*co*-Phe) micelles (NSM) showed a better synergistic effect against colon cancer compared with free Sor and Met treatment. Figure 1 shows the preparation process of Sor and Met co-loaded micelles and the mechanisms against CRC.

## Materials and methods

The materials, synthesis of mPEG-b-P(BLG-*co*-Phe) and mPEG-b-P(Glu-*co*-Phe) copolymers, preparation of mPEG-b-P(Glu-*co*-Phe)/Sor/Met micelles, characterizations of copolymers, NSM stability, *in vitro* drug release, cytotoxicity assays, and cellular uptakes are shown in the Supplementary File.

### Animal study

This study was approved by the Jilin University Animal Center (KT202002042). BALB/c mice (male, 8–12 weeks) and Sprague-Dawley rats (male, 180–200 g) were used and bought from Jilin University.

The subcutaneous animal model was established by injecting CT26 cells (0.1 mL, 100 × 10^4^ mL^−1^) into the right flanks of BALB/c mice. When the tumors were about 300 mm^3^, the animals were divided into three groups with six animals in each group, i.e., normal saline (control), free Sor and Met (SM), and NSM at Sor dose of 10 mg kg^−1^ and Met dose of 40 mg kg^−1^. Then, 100 μL normal saline, SM solution, or NSM solution was applied through the tail vein five times every 3 days.

### *In vivo* biodistribution

Twelve mice in the subcutaneous colon cancer mice model with a tumor volume of about 300 mm^3^ were selected and were divided into NSM and SM groups. The mice in the SM group were treated with SM saline solution *via* tail vein injection with Sor dose of 20 mg kg^−1^ and Met dose of 80 mg kg^−1^. The mice in the other group were treated with NSM solution with the equivalent amounts of Sor and Met to those in NSM solution. The mice were euthanized at 6 or 12 h after injection. The tumor tissues and other major organs were resected. The Sor and Met in different tissues were determined with the HLPC method.

### Pharmacokinetic detections

Sprague-Dawley rats were divided into NSM and SM groups (*n* = 3). NSM or SM solutions (2 mL) with equivalent Sor dose of 20 mg kg^−1^ and Met dose of 80 mg kg^−1^ were injected *via* tail vein. Blood samples were collected at different time points. The Sor and Met concentrations were analyzed with the HLPC method.

### *In vivo* antitumor efficiency assessment

The largest diameter (L) and smallest diameter (S) of tumors were measured every day, and the tumor volume was calculated with Equation (1).


(1) $${\text{V (mm}}^{\text{3}})=\frac{L\times S^{2}}{2}$$

After 14 days post-treatment, all the mice were euthanized. The tumor growth rate (TGR) was calculated by the ratio of the tumor volume at 14 days post-treatment and the tumor volume before treatment. Blood samples were collected and the levels of ALT, AST, CK-MB, BUN, and D-Lac were analyzed by the ELISA method. The tumor weight of each sample was recorded.

### Histopathological study

The tumor tissues and other major organs were stained with hematoxylin and eosin (H&E). The immunohistochemical assays were also applied to evaluate ERK, p-ERK, and cyclin D1 levels in tumor tissues. The positive cells were stained brown-yellow, and the relative positive area was analyzed by Image J (National Institutes of Health, Bethesda, Maryland, USA).

### Statistical analyses

One-way ANOVA and Student's *t*-test were used. *P* < 0.05 indicated statistically significant, and *P* < 0.01 and *P* < 0.001 indicated highly statistically significant.

## Results and discussions

### Preparation of NSM and characterizations

Figure 1 shows the NSM preparation and the mechanisms for the CRC therapy.

The FT IR and GPC analyses indicated the successful development of mPEG-*b*-P(Glu-*co*-Phe) copolymers (Supplementary Figure S1). Relevant results are shown in the Supplementary File.

As shown in Figure 1, NSM is prepared in an aqueous solution, utilizing the electrostatic interaction between glutamic acid carboxyl group and the Met amino group. Sor is loaded within the nanocarrier by a simple physical embedding method. The mPEG-*b*-P(Glu-*co*-Phe) copolymers self-assembled in aqueous solutions and entrap Sor and Met within micelles. The electrostatic interactions between the drugs and polymers would benefit the release of drugs. The electrostatic interactions will be damaged in the acidic environment within tumor tissues, thus resulting in drug-releasing (6). As shown in Table 1, when the Sor and Met feeding ratios were 11.2 and 29.4%, satisfactory DLCs and DLEs of Sor and Met could be obtained. A higher drug feeding ratio resulted in slightly increased DLC, while DLE was decreased remarkably. As a result, the DLCs of 4.9 and 11.4% of Sor and Met, respectively, were applied to obtain a rationale DLC and a high DLE. The DLEs of Sor and Met of NSM were 74.8 and 85.4 wt.%, respectively. The polymeric chemotherapy drug delivery systems were acceptable for DLC ranging from 1 to 20% (26).

**Table 1:** DLC and DLE of Sor and Met of NSM.

**Feed ratio (w/w/w)** **(Polymer/Sor/Met)**	**DLC Sor (%)**	**DLE Sor (wt.%)**	**DLC Met (%)**	**DLE Met (wt.%)**
45:05:15	2.1	77.2	6.6	86.6
40:08:20	4.9	74.8	11.4	85.4
35:15:25	12.3	54.8	18.8	63.6
30:20:30	20.2	46.4	25.6	51.2

**Figure 2:**
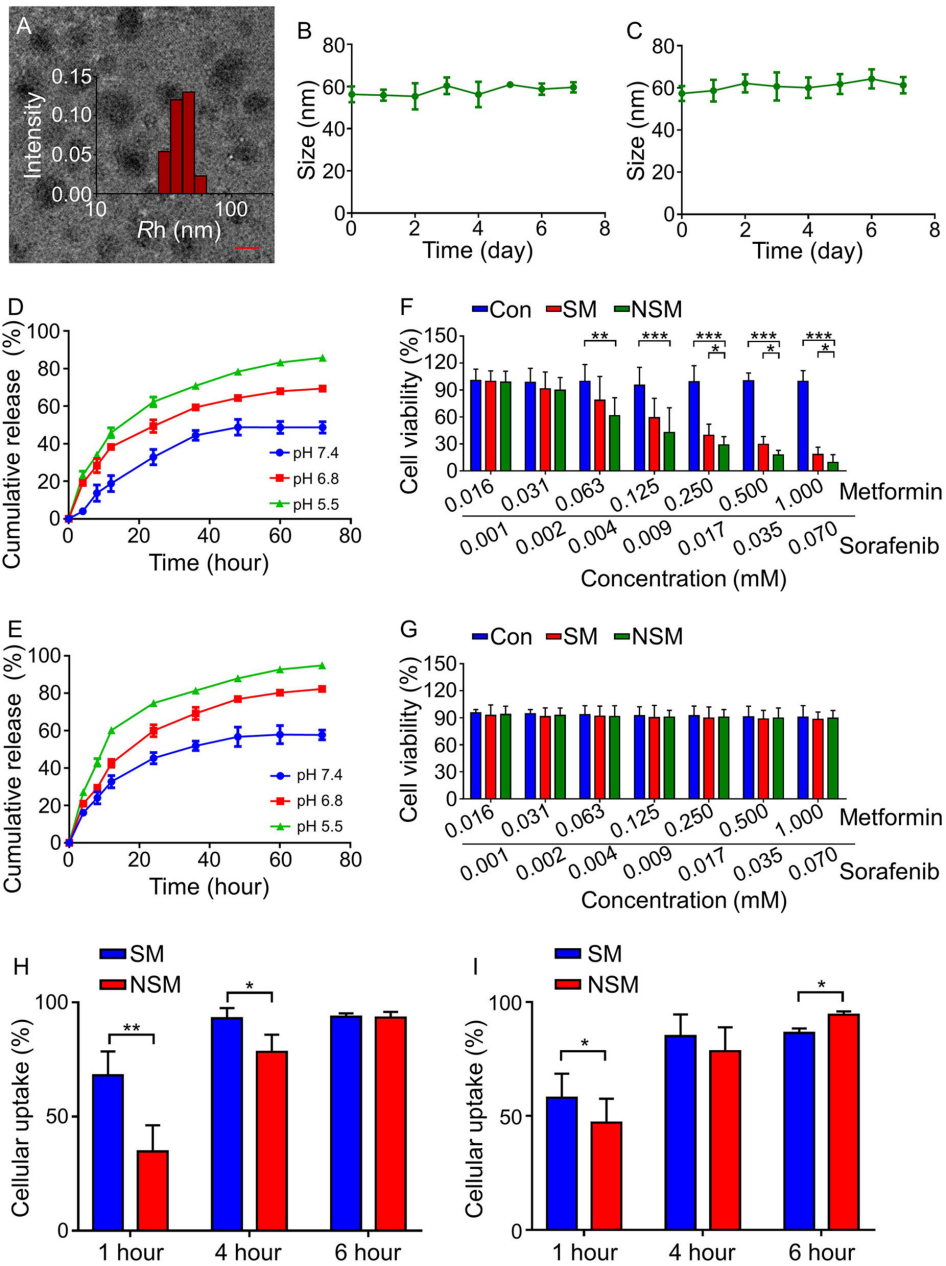
Characterizations of mPEG-*b*-P(Glu-*co*-Phe) micelles. **(A)** TEM and DLS analyses. NSM stability in **(B)** PBS and **(C)** BSA solution. Release profiles of **(D)** Sor and **(E)** Met in PBS solution at different pH values. *In vitro* cytotoxicity analyses on **(F)** H22 cells and **(G)** HLL-5 cells at different Met and Sor concentrations in different groups. Cellular uptakes of **(H)** Sor and **(I)** Met of SM and NSM after incubation with H22 cells for 1, 4, and 6 h. Scale bar = 50 nm. *, **, *** represent *P* < 0.05, *P* < 0.01, and *P* < 0.001, respectively.

The micelles' morphology is observed under TEM examination (Figure 2A), demonstrating that NSM is homogeneously spherical with narrow size distribution. The size distributions of NSM were evaluated with DLS in this study. The size distribution results are similar to TEM, in which all the micelles show a pretty narrow distribution (Figure 2A). The average diameter of NSM is 67.3 ± 8.9 nm as observed by DLS analysis. The diameter of nanoparticles of 100 nm is suitable to enhance the permeability and retention (EPR) effect (27, 28). The diameter of NSM is slightly smaller than 100 nm, and the nanoparticles of this size are also suitable for tumor therapy (6).

The stability of drug delivery systems is crucial in drug delivery. As shown in Figures 2B,C, the incubation time lasts for 7 days, but no obvious size changes are observed in both incubation mediums. The NSM shows excellent stability in the neural environment in this study.

### *In vitro* drug release

The release profiles of Sor and Met were studied in PBS solution at pH 7.4, 6.8, and 5.5 at 37°C. The amounts of released drugs were examined with HPLC. The release behavior of Sor was similar to that of Met in which three different release conditions could be observed. As shown in Figures 2D,E, a rapid release happened in the first 24 h, followed by a slower release at 24–48 h and a sustained release at 48–72 h. There are 48.7 ± 3.1% and 57.7 ± 2.6% amounts of Sor and Met released after 72 h of incubation at pH 7.4, respectively (Figures 2D,E). About 50 and 40% amounts of Sor and Met are not released because the electrostatic interaction within the micelles was not seriously weakened at pH 7.4 possibly (29). Besides, hydrophobic phenylalanine units enhanced the stability of micelles, resisting the micelles' dissociation (6). As a result, there were still some drugs not released from the inner core of the micelles. The release profiles of Sor and Met are pH responsive with more Sor and Met released in an acidic environment. There are 69.4 ± 2.7% and 85.8 ± 1.9% amounts of Sor released after 72 h of incubation at pH 6.8 and 5.5, respectively (Figure 2D). The amounts of released Met are 82.3 ± 2.6% and 94.9 ± 2.5% after 72 h incubation at pH 6.8 and 5.5, respectively (Figure 2E). The release of Sor and Met happened simultaneously, but Met was released a little faster than that of Sor at the same pH value. The increased acidity of the tumor microenvironment could facilitate the disruption of electrostatic interaction and promote the instability of micelles, thus facilitating more drug release for tumor therapy (6).

### *In vitro* cytotoxicity and cellular uptakes

After 48 h, the cell viabilities of CT26 cells are all above 90% (Figure 2F), indicating that the copolymers possess good compatibility and low cytotoxicity. As for the drug-loaded MTT assay, both SM and NSM exhibit dose-dependent cytotoxicity effects toward CT26 cells (Figure 2F). When the concentration of Met is more than 0.063 mM, and Sor is more than 0.004 mM, the cell viability is lower in the NSM group than that in the SM group (*P* < 0.05) and control group (*P* < 0.001), demonstrating that NSM possesses stronger cell proliferation inhibition efficiency than SM. However, NSM did not show severe cytotoxicity to normal human intestinal mucosa endothelial cells HIEC. The viability of HIEC cells was all above 85% and there was no significant difference in cell viability among all groups (Figure 2G). The *in vitro* cytotoxicity was repeated three times.

Efficient cellular uptakes of drugs can increase antitumor activity. The cellular uptakes of Sor (Figure 2H) and Met (Figure 2I) in SM and NSM groups are evaluated with the HPLC. The general cellular uptakes of Sor and Met are higher in the SM group than in the NSM group at 1 h, which may be attributed to the fact that free Met and Sor can be rapidly uptaken by CT26 cells during the first hour. At 4 h, the cellular uptake of Sor is slightly higher in the SM group than that in the NSM group (^*^P < 0.05). However, there is no significant difference in the cellular uptake of Met between the two groups at 4 h. There is no difference in the Sor cellular uptake at 6 h between SM and NSM groups. The cellular uptake of Met is higher in the NSM group than that in the SM group at 6 h (^*^P < 0.05). This may be because free SM and NSM may have different cellular uptake methods, and mPEG-b-P(Glu-co-Phe) may increase the ability of Sor and Met to enter the cell. The cellular uptakes of Met and Sor in the NSM group are all above 90% at 6 h. The high cellular uptakes of Sor and Met could benefit the synergistic chemotherapeutic effects against CRC.

### Biodistribution studies

The biodistributions of Met and Sor in different tissues were detected with the HPLC method in this study.

**Figure 3:**
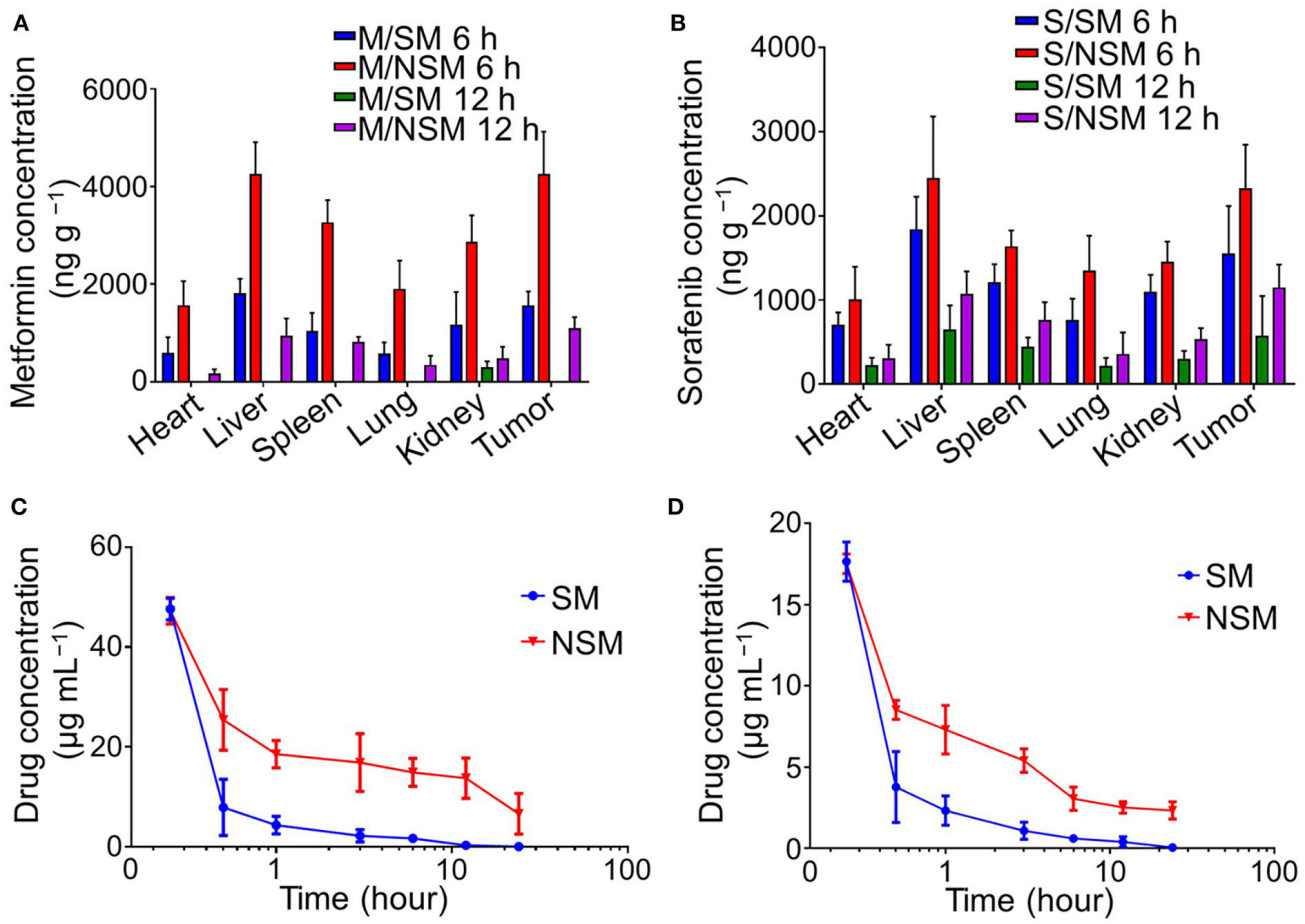
Biodistribution and pharmacokinetics studies of Met and Sor. Biodistribution of **(A)** Met and **(B)** Sor in subcutaneous colon cancer mice model. Plasma pharmacokinetics of **(C)** Met and **(D)** Sor in SM and NSM groups.

The amounts of Met and Sor were at higher levels in the tissues at 6 h post-injection than that at 12-h post-injection. There were more Met and Sor accumulated in the NSM groups than in the SM group. Met and Sor are mainly located in liver and tumor tissues in the subcutaneous colon cancer mice model (Figures 3A,B). At 12-h post-injection, Met only accumulates in the kidney and disappears from tumor tissues in the SM group. In the NSM group, amounts of Met in tumor tissues can also be observed. The amounts of Met and Sor are statistically higher in tumor tissues after NSM treatment than after SM injection, indicating that the NSM can target the tumor site. The sustained release of Met and Sor from the micelles within tumors contributed to the accumulation of drugs.

### Pharmacokinetic detections

Plasma pharmacokinetics of Met and Sor in SM and NSM are evaluated with HPLC post-intravenous administration (Figures 3C,D). The Met and Sor concentrations in the SM group decrease dramatically in the first 30 min and slowly decrease after that. The burst drug concentrations decrease was not evident in the NSM group, and the drug concentrations decreased much slower than that in the SM group. As a result, the blood circulation time of NSM could be significantly enhanced compared to SM. The Met and Sor clearance in mPEG-*b*-P(Glu-*co*-Phe) micelles decreased due to the increased stability of polymeric micelles and sustained drug delivery possibly.

### *In vivo* anticancer efficiency

**Figure 4:**
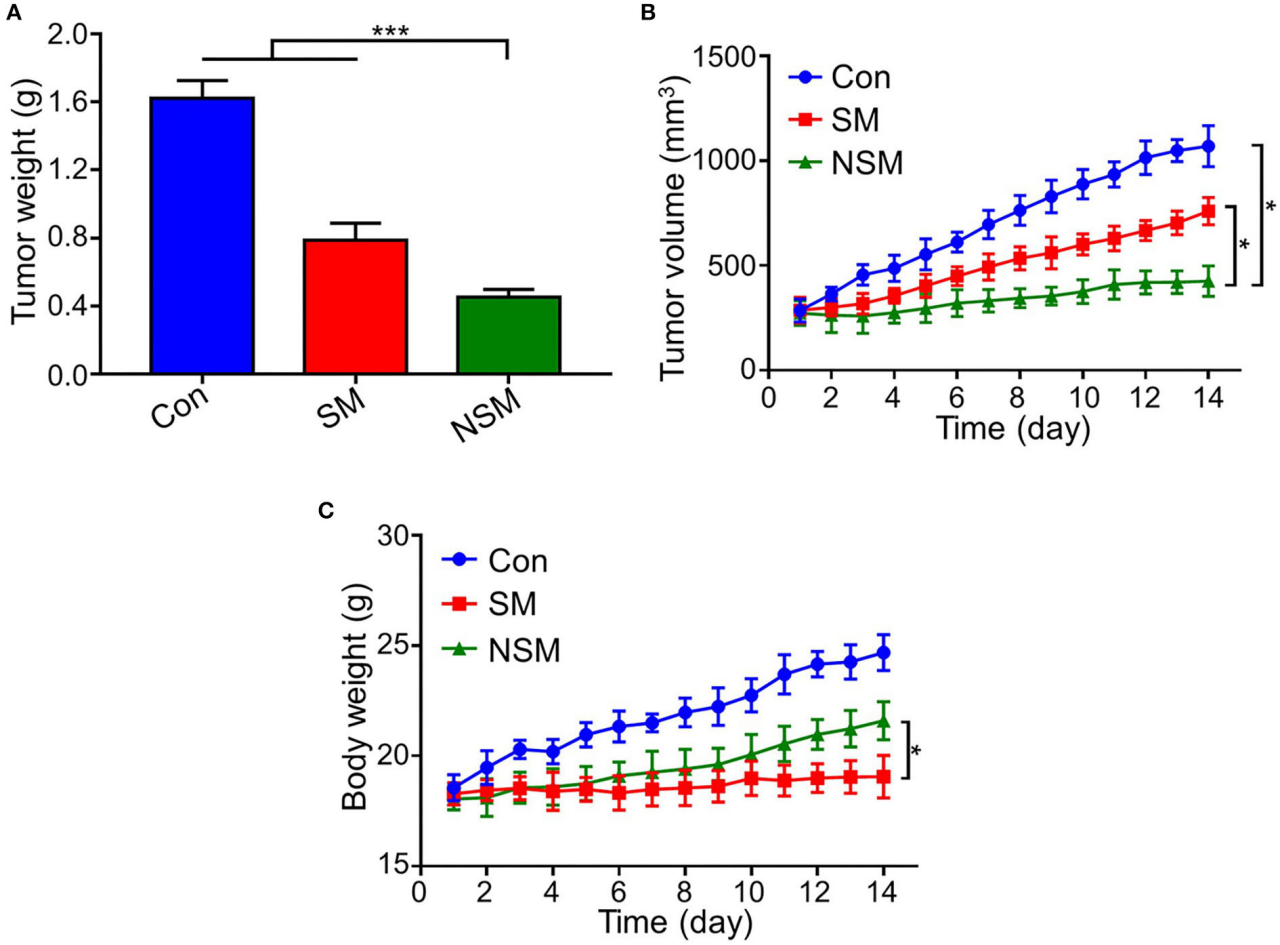
Antitumor efficacy of NSM in subcutaneous colon cancer mice model. **(A)** Tumor weight. **(B)** Tumor volume. **(C)** Body weight of mice. * and *** represent *P* < 0.05 and *P* < 0.001, respectively.

The subcutaneous colon cancer mice model was performed to evaluate the anticancer efficiency of NSM. After the sacrifice of mice, the tumors are carefully resected to further assess the *in vivo* antitumor efficiency, and the tumor weights are also recorded. The tumor weight is the least in the NSM group, and the difference is significant between SM and NSM groups (*P* < 0.001) (Figure 4A). Consistent with the tumor weight results, the tumor volume is also the least in the NSM group (Figure 4B). There is a significant difference between SM and NSM groups in tumor volume. In addition, the TGRs of NSM, SM, and Control groups are 4.13 ± 0.61, 2.42 ± 0.35, and 1.59 ± 0.22, respectively. NSM group presents a higher TGR compared with SM group (*P* < 0.05) and Control group (*P* < 0.01). As shown in Figure 4C, there was no obvious weight loss or increase in body weight in the SM group, which indicates that even the free SM treatment seems to be well-tolerated and causes no weight loss. The mice show an evident increase in body weight in the control group and the NSM group. The body weight of the control group increased gradually and was the highest compared with the other two groups. This may also be explained by the growing tumor and little drug toxicity effect. The difference is significant in body weight between the SM and NSM groups (*P* < 0.05). The general body conditions of animals are good after NSM treatment due to small toxicity. The results demonstrated that NSM possessed higher tumor inhibition efficiency over SM treatment. This is because of the increased accumulation of NSM and the fast release of Met and Sor from the micelles within the tumor tissues possibly.

### Biochemical analyses

Biochemical analyses were applied to evaluate the general conditions of major organs. Besides, the toxicity of Met was evaluated by testing D-Lac levels, which were inclined to induce lactic acidosis (30).

**Figure 5:**
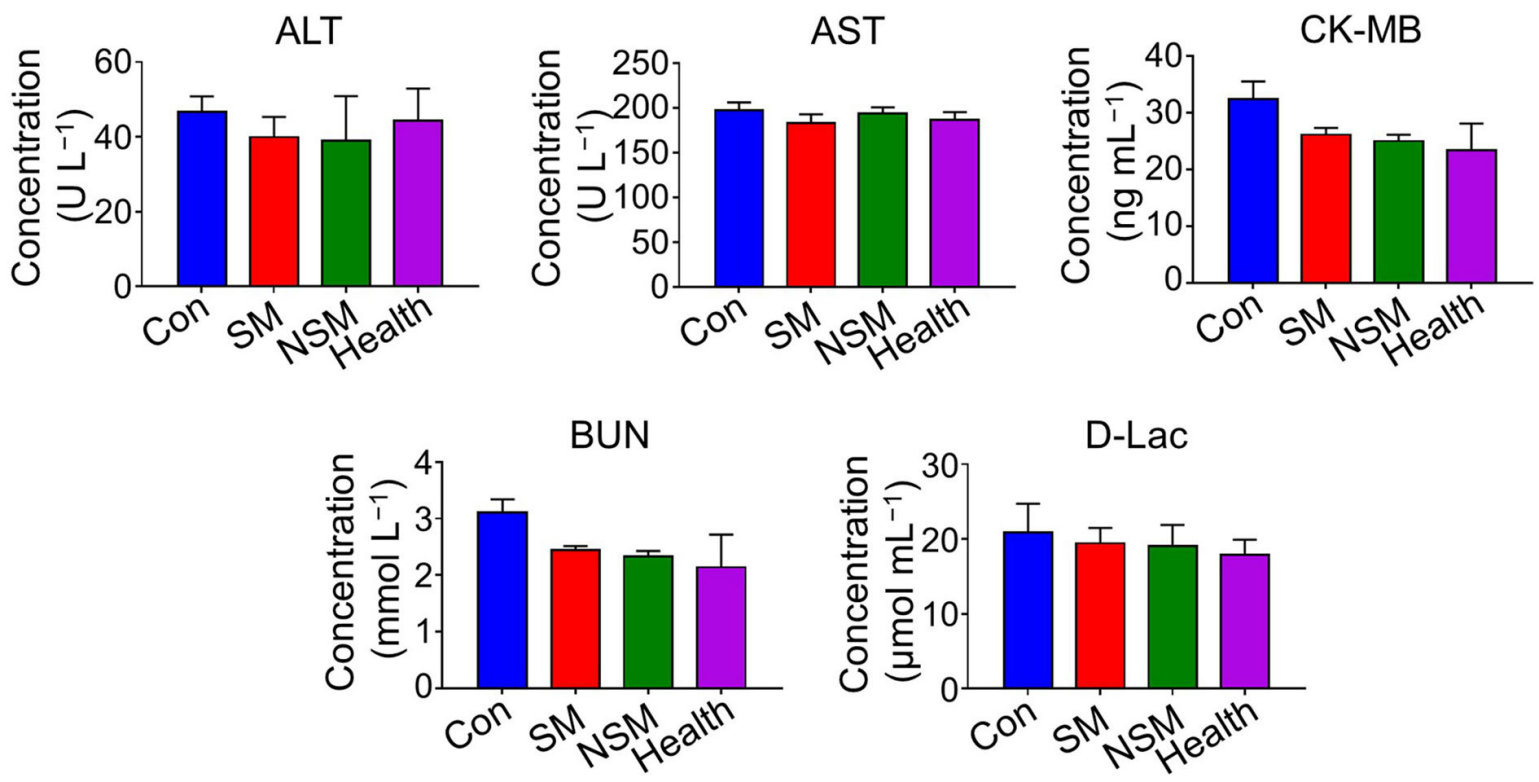
Biochemical analyses in subcutaneous colon cancer mice model.

Figure 5 shows the biochemical analyses of the subcutaneous colon cancer mice model. There was no statistical difference in ALT, AST, CK-MB, BUN, and D-Lac levels among all the groups, indicating that obvious liver, heart, and kidney injuries are not caused by the NSM and SM treatment. Also, the application of Met in free SM solution or NSM micelles does not increase D-Lac levels or induce lactic acidosis.

### Histopathological evaluations

**Figure 6:**
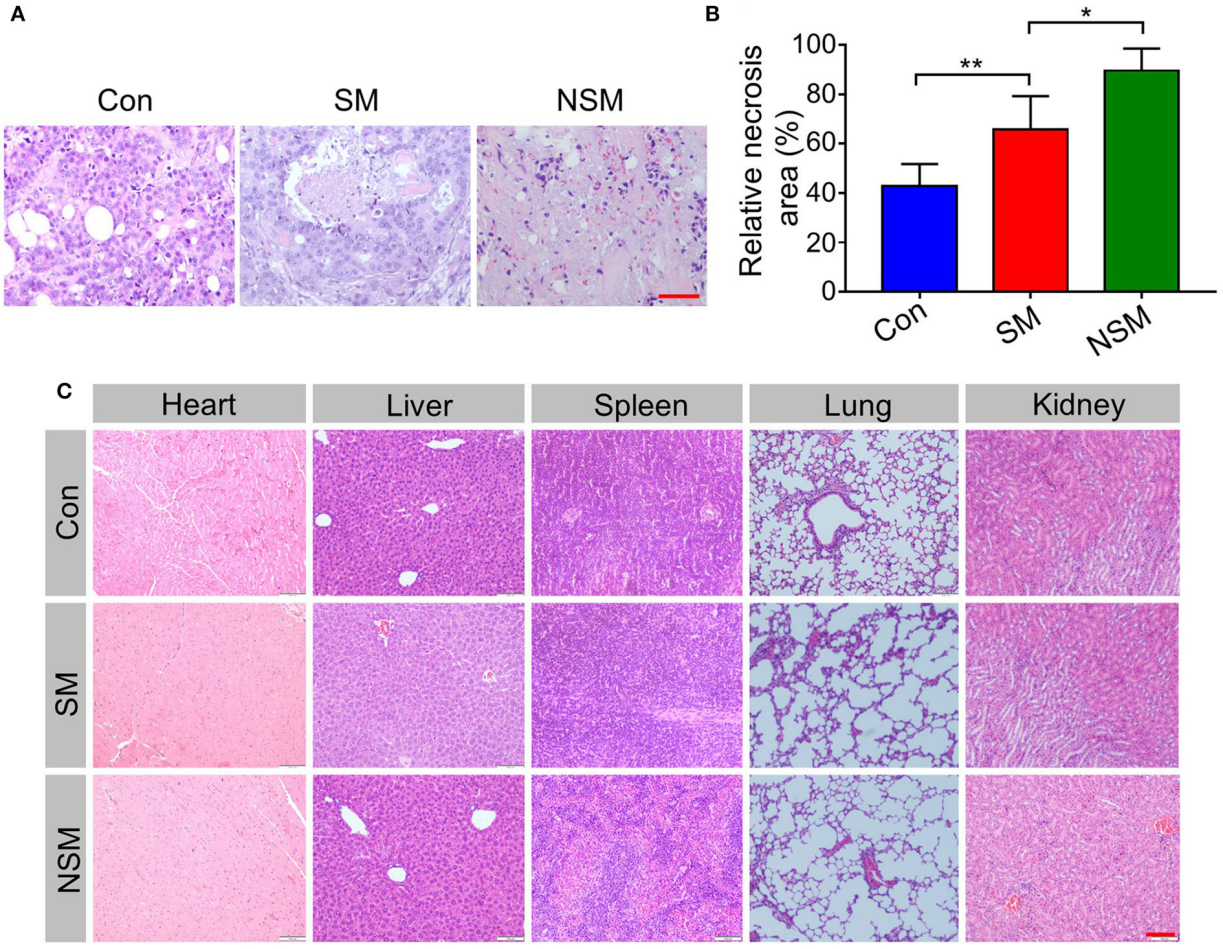
Histopathological analysis. **(A)** H&E staining and **(B)** relative necrosis area of tumor tissues. **(C)** H&E analysis of major organs. Scale bars = 100 μm. * and ** represent *P* < 0.05 and *P* < 0.01, respectively.

H&E analysis of tumor sections was performed to assess the tumor inhibition efficiency of NSM (Figure 6A). The relative necrosis area of the tumor was analyzed with the Image J software. The necrosis area is small in the control group, indicating rapid proliferation of tumor cells (Figure 6B). However, various necrosis degrees can be found in SM and NSM groups. NSM shows the least necrosis tumor area, and there is a significant difference between SM and NSM groups (*P* < 0.05) (Figure 6B).

The biological values of ALT, AST, CK-MB, BUN, and D-Lac are first tested in this study. The results do not reveal apparent damage in normal organs. H&E analysis of major organs is performed to analyze the security of NSM further. In the control group, the H&E staining of organs shows the normal histological structure (Figure 6C). No obvious pathological changes are found in SM and NSM groups, indicating the high security of NSM in CRC treatment.

### Immunohistochemical analyses

The ERK, p-ERK, and cyclin D1 levels were analyzed to reveal the anticancer mechanism of NSM.

**Figure 7:**
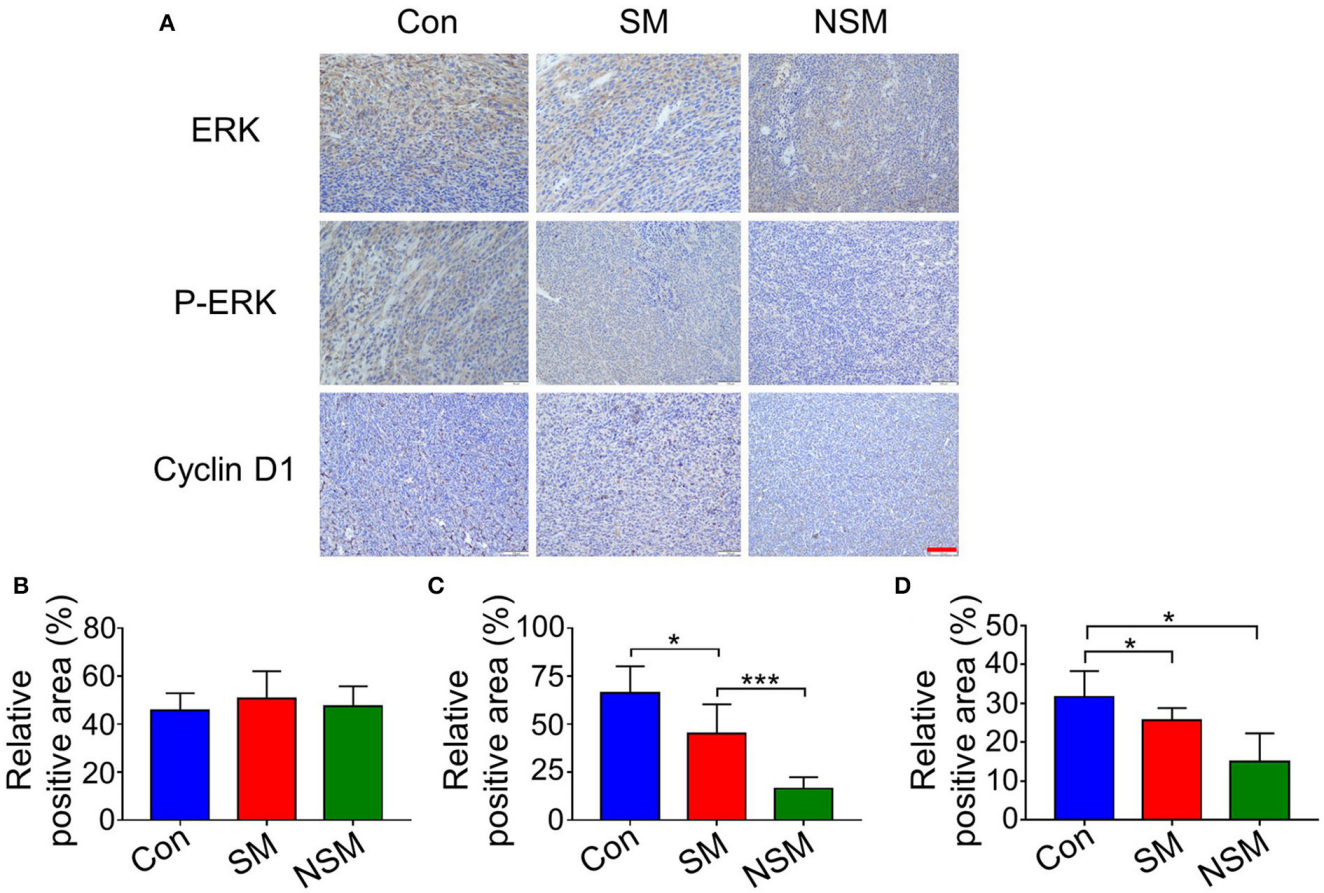
Immunohistochemical analyses of tumor tissues. **(A)** Immunohistochemical staining of ERK, p-ERK, and Cyclin-D1 in tumor tissues. Relative positive areas of **(B)** ERK, **(C)** p-ERK, and **(D)** Cyclin-D1. Scale bar = 100 μm. * and *** represent *P* < 0.05 and *P* < 0.001, respectively.

The MAPK/ERK pathway is one of the key pathways for solid tumor development. The inhibition of the MAPK/ERK pathway could not phosphorylate ERK, reducing the proliferation of tumor cells (31, 32). Immunohistochemical studies first test the expression levels of ERK and p-ERK. All three groups show similar amounts of positive cells for ERK evaluation. However, the most and least amounts of p-ERK are found in the control and NSM groups, respectively (Figure 7A). The immunohistochemical results are confirmed with the semi-quantitative analyses (Figures 7B,C).

Cyclin D1 is one of the most important regulators of the cell cycle (33). The upregulation of cyclin D1 could lead to cell cycle disorders and highly promote cell proliferation (33). The expressions of cyclin D1 are tested to determine whether NSM treatment was associated with cell cycle arrest. The immunohistochemical staining and semi-quantitative analyses show that the expressions of cyclin D1 are most inhibited in the NSM group (Figures 7A,D).

The above results demonstrate that NSM mainly performs its tumor inhibition efficiency through downregulating the expressions of p-ERK and cyclin D1, thus inhibiting the MAPK/ERK pathway and influencing the cell cycle. As a result, the proliferation of tumors can be prevented.

## Conclusion

In this study, an mPEG-*b*-P(Glu-*co*-Phe) copolymer-based drug delivery system was developed. Sor and Met were loaded in the mPEG-*b*-P(Glu-co-Phe) micelles to achieve the chemotherapeutic effect. NSM can be targeted to cancer cells and release Sor and Met rapidly within tumors. A subcutaneous colon cancer mice model was developed to assess the anticancer efficacy of NSM. NSM can inhibit tumor proliferation through the synergistic effect of Sor and Met on blocking the MAPK/ERK pathway and arresting the cell cycle of colon cancer cells. All these results demonstrated the superiority of Sor/Met loaded-mPEG-*b*-P(Glu-*co*-Phe) micelles in the treatment of CRC.

## Data availability statement

The raw data supporting the conclusions of this article will be made available by the authors, without undue reservation.

## Ethics statement

The animal study was reviewed and approved by the Experimental Center of Jilin University.

## Author contributions

XZ wrote the manuscript. LC, GX, and HH performed the study. HH analyzed the data. TL and HZ revised the manuscript. All authors contributed to the article and approved the submitted version.

## Conflict of interest

The authors declare that the research was conducted in the absence of any commercial or financial relationships that could be construed as a potential conflict of interest.

## Publisher's note

All claims expressed in this article are solely those of the authors and do not necessarily represent those of their affiliated organizations, or those of the publisher, the editors and the reviewers. Any product that may be evaluated in this article, or claim that may be made by its manufacturer, is not guaranteed or endorsed by the publisher.
