# Supplementary material for: Co-delivery of sorafenib and metformin from amphiphilic polypeptide-based micelles for colon cancer treatment
Source: Front Med (Lausanne). 2022 Oct 11;9:1009496. doi: 10.3389/fmed.2022.1009496 (PMC9592705; doi:10.3389/fmed.2022.1009496)
Supplement: Supplementary file 1 [file Data_Sheet_1.pdf]

Co-delivery of sorafenib and metformin from amphiphilic polypeptide-based micelles for colon cancer treatment

Xiaohui Zhang[1,2], Lanqing Cao[3], Guangmeng Xu[1], Hongyu He[4], Hongyu Zhao[5,*], Tongjun Liu[1,*]

[1]Department of General Surgery, The Second Hospital of Jilin University, Changchun, Jilin, China

[2]Department of Thyroid, Breast and Hernia Surgery, Affiliated Hospital of Inner Mongolia University for the Nationalities, Tongliao, Inner Mongolia, China

[3]Department of Pathology, The Second Hospital of Jilin University, Changchun, Jilin, China

[4]Operating Theater and Department of Anesthesiology, The Second Hospital of Jilin University, Changchun, Jilin, China

[5]Gastroenterology and Center of Digestive Endoscopy, The Second Hospital of Jilin University, Changchun, China

Correspondence:

Hongyu Zhao, Gastroenterology and Center of Digestive Endoscopy, The Second Hospital of Jilin University, Changchun No.218 Ziqiang Street, China. Email: zhaohy14@mails.jlu.edu.cn

Tongjun Liu, Department of General Surgery, The Second Hospital of Jilin University, Jilin, Changchun No.218 Ziqiang Street, China. Email: tongjunliu@163.com

Materials and methods

Materials

Methoxy poly(ethylene glcol) (mPEG, M_n = 5000) was purchased from Sigma-Aldrich (Shanghai, P. R. China). mPEG-NH_2 and BLG NCA were prepared, according to a previous study (Ding et al., 2011). L-Phenylalanine-N-carboxyanhydride (Phe-NCA, 98%) was bought from Beijing Huafeng United Technology Co., Ltd. (Beijing, P. R. China). Sor and Met were bought from Beijing Pharmacy (Beijing, P. R. China). Fetal bovine serum

(FBS) and RPMI 1640 medium were bought from Gibco (Grand Island, NY, USA). The dimethyl sulfoxide (DMSO), 3-(4,5-dimethyl-thiazol-2-yl)-2,5-diphenyl tetrazolium bromide (MTT), and Ethyl Alcohol 95% were bought from Sigma-Aldrich (Shanghai, P. R. Alanine transaminase (ALT), aspartate aminotransferase (AST), creatine kinase MB isoenzyme (CK-MB), blood urea nitrogen (BUN), and D-lactate (D-Lac) ELISA kits were purchased from Longton Co. Ltd. (Shanghai, P. R. China). The primary antibodies of ERK, p-ERK, and cyclin D1 were purchased from Abcam Reagent Co., Ltd. (Cambridge, MA, USA).

Synthesis of mPEG-b-P(BLG-co-Phe) copolymers

mPEG-*b*-P(BLG-*co*-Phe) was synthesized through ring-opening polymerization (ROP) (Lv et al., 2013). mPEG-NH_2 (5 g, 1 mmol) was first added into an ampoule (250 mL) followed by azeotropic dehydration with toluene

Dry DMF was used to dissolve the above anhydrous mPEG-NH_2, and then BLG-NCA (3.949 g, 15 mmol) and Phe-NCA (0.955 g, 5 mmol) were added into the solution. After stirring for three days, 500 mL anhydrous ether was used to precipitate the mPEG-*b*-P(BLG-*co*-Phe) copolymers. The copolymers were finally dried under vacuum.

Synthesis of mPEG-b-P(Glu-co-Phe) copolymers

mPEG-*b*-P(BLG-*co*-Phe) copolymers (5 g) were dissolved in dichloroacetic acid (50 mL), and HBr/acetic acid (15 mL, 33 wt.%) was added. Then the solution was stirred for one hour, precipitated by excessive ice anhydrous ether, and then dried under vacuum. The precipitate was dissolved in DMF, dialyzed, and dried to obtain mPEG-*b*-P(Glu-*co*-Phe).

Preparation of mPEG-b-P(Glu-co-Phe)/Sor/Met micelles

To prepare the Met and Sor co-loaded micelles, Met·HCl, Sor, and mPEG-b-P(Glu-co-Phe) were dissolved in DMF, and added to deionized water and stirred for 24 hours. The pH value of deionized water was adjusted to 7.0–7.5 with 0.1 M NaOH. Free Sor and Met were removed by dialysis for 12 hours. The product was lyophilized to obtain the nanosized mPEG-*b*-P(Glu-co-Phe) Sor/Met-loaded micelles (NSM). The amount of Sor and Met within micelles was determined by high-performance liquid chromatography (HPLC) equipped with a multiple wavelength ultraviolet-visible (UV-vis)

detector (Waters 2475 Multi I Fluorescence Detector, USA). During the chromatographic procedure, Waters e2695 Separation column (4.6 mm × 250 mm, 5 μm) was used, and the mixture of 20 mM ammonium acetate, and acetonitrile (40:60 v/v) was the mobile phase. The flow rate of the mobile phase was 1 mL min^{-1}. So/Met loaded-micelles were suspended in methanol and then stirred for 5 hours. The solution was filtrated twice, and the supernatant was added to the column. The UV detector of Sor and Met was set at 255 nm and 233 nm, respectively. Sor and Met in methanol were in the concentration range of 0.2 – 200 μg mL^{-1}. The drug loading capacity (DLC) and drug loading efficiency (DLE) were calculated with Eq. 1 and Eq. 2.

$$\text{DLC (\%)} = \frac{\text{Amount of loaded drug}}{\text{Amount of drug-loaded micelle}} \times 100\% \quad (1)$$

$$\text{DLE (\%)} = \frac{\text{Amount of loaded drug}}{\text{Total amount of feeding drug}} \times 100\% \quad (2)$$

Characterizations

mPEG-*b*-P(BLG-co-Phe) and mPEG-*b*-P(Glu-*co*-Phe) were characterized by Fourier-transform infrared (FT IR) and gel permeation chromatography (GPC). Through potassium bromide, FT IR was performed on a Bio-Rad Win-IR instrument (Bio-Rad Laboratories Inc., Cambridge, MA, USA). GPC analyses were conducted on a Waters GPC system with a Waters Styragel HT6E column and a Waters 2414 refractive index detector. The DMF which contained 0.01 M lithium bromide (LiBr) acted as the eluent, and the flow rate was 1.0 mL min^{-1}.

The morphology of NSM was observed by transmission electron microscope (TEM; JEOL; Tokyo, Japan). The diameter changes of NSM were analyzed by dynamic laser scattering (DLS) on a Wyatt QELS instrument (Wyatt Technology Corp., Santa Barbara, CA, USA).

NSM stability

The stabilities of NSM in phosphate-buffered saline (PBS) solution and 25.0 mg mL^{-1} bovine serum albumin (BSA) solution of pH 7.4 were determined by DLS at pre-set time intervals at room temperature.

In vitro drug release

The *in vitro* Sor and Met release study was conducted using the dialysis method. Briefly, 1.0 mg NSM was dissolved in 10.0 mL PBS solution (pH 7.4, 6.8, and 5.5), and then the solution was introduced into an end-sealed dialysis tubing (MWCO 35 kDa) in 100.0 mL PBS solution. The solution was stirred at 70 rpm and 37°C. Control experiments were performed by placing free Sor or Met solution in dialysis tubing (MWCO 35 kDa) in 100.0 mL PBS solution (pH 7.4). At pre-set time intervals, 2.0 mL dialysate was collected and replaced by the same amount of fresh incubation medium. Sor and Met releases were determined through the HPLC method mentioned above.

Cytotoxicity assays

Cytotoxicity of NSM on CT26 cell lines and normal human intestinal mucosa endothelial cells HIEC was detected using the MTT assay. CT26 or HIEC cells were cultured in 96 microtiter plates with 12000 cells/well. After 24-hour culture, NSM or free Sor and Met (SM) or blank micelles were added into the original medium. The concentrations of Sor in NSM or SM medium solution ranged from 0.001 to 0.070 mM. The concentrations of Met ranged from 0.016 to 1.000 mM. After 48-hour culture, 5 mg/mL MTT was added to each well, and the cells were cultured for another 4 hours. Finally, the medium was withdrawn and 150 μL acidified isopropanol was added to each well. A Bio-Rad 680 microplate reader (Hercules, CA, USA) was used to measure the absorbance of the final solution at 570 nm. The cell viability was calculated with Eq. 3.

$$\text{Cell viability (\%)} = \frac{A_{\text{sample}}}{A_{\text{control}}} \times 100\% \quad (3)$$

A_{sample} and A_{control} are the absorbances of the solution in sample and control wells, respectively.

Cellular uptakes

The cellular uptakes of Sor and Met were detected by HLPC. CT26 cells (10×10^4 cells/well) were seeded in 6-well plates and cultured in RPMI-1640 medium overnight in a 5% CO_2 environment at 37°C. NSM or SM was then added to each well, and the final concentrations of Sor and Met were 10 μmol mL^{-1} and 23.5 μmol mL^{-1}, respectively. After the culture 1, 4, or 6 hours, the CT26 suspension was centrifuged at 3000 rpm for 10 min. The supernatant was discarded, and CT26 cells were washed with PBS three times. The

cells were then stored in a –80°C environment for one hour and thawed at room temperature for 30 min. The freezing and thawing were repeated three times. The final solution was centrifuged at 3000 rpm for 10 min, and the supernatant was collected. The uptakes of Sor and Met were determined through the HPLC method mentioned above.

Results

mPEG-*b*-P(BLG-co-Phe) copolymers were prepared by the ROP of BLG-NCA and Phe-NCA. The FT IR spectra of mPEG-*b*-P(Glu-*co*-Phe) and mPEG-*b*-P(BLG-*co*-Phe) are shown in Figure S1A. The signals in 1150 cm^{-1}, 1505 cm^{-1}, and 1604 cm^{-1} in the FT IR spectra of mPEG-*b*-P(BLG-*co*-Phe) are assigned to the stretching vibrations of C–O–C bond of PEG, C–N, and C=O in amide bond of BLG, respectively. For mPEG-*b*-P(Glu-*co*-Phe), the absorption at 1149 cm^{-1} belonged to the C–O–C bond of PEG, and the absorption at 1524 cm^{-1} (vC(O)–NH) and 1627 cm^{-1} (vC=O) belonged to the amide bond on the polypeptide backbone. GPC analyses show a narrow molecular weight distribution of mPEG-*b*-P(BLG-*co*-Phe) (Figure S1B).

References

Ding, J., Zhuang, X., Xiao, C., Cheng, Y., Zhao, L., He, C., Tang, Z., and Chen, X. (2011). Preparation of photo-cross-linked pH-responsive polypeptide nanogels as potential carriers for controlled drug delivery. *Journal of Materials Chemistry* 21**,** 11383-11391.

Lv, S., Li, M., Tang, Z., Song, W., Sun, H., Liu, H., and Chen, X. (2013). Doxorubicin-loaded amphiphilic polypeptide-based nanoparticles as an efficient drug delivery system for cancer therapy. *Acta Biomaterialia* 9**,** 9330-9342.

Figure legends

Figure S1. Characterizations of mPEG-*b*-P(BLG-co-Phe) and mPEG-*b*-P(Glu-*co*-Phe) copolymers. (A) FT IR spectra, and (B) GPC analyses of mPEG-*b*-P(Glu-*co*-Phe) and mPEG-*b*-P(BLG-*co*-Phe).
