# Supplementary figures and images for: Co-delivery of sorafenib and metformin from amphiphilic polypeptide-based micelles for colon cancer treatment
Source: Front Med (Lausanne). 2022 Oct 11;9:1009496. doi: 10.3389/fmed.2022.1009496 (PMC9592705; doi:10.3389/fmed.2022.1009496)

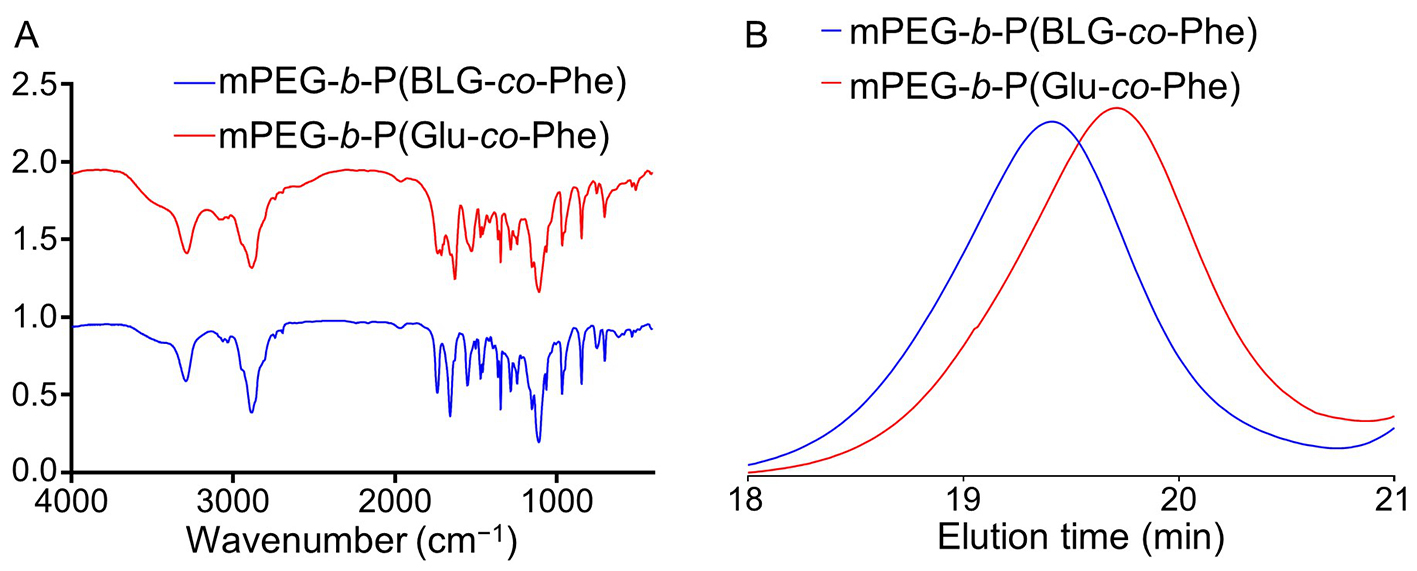

Supplement: Supplementary file 2 [file Image_1.JPEG]
